# Development of a Smartphone App for Women Living With Gestational Diabetes Mellitus: Qualitative Study

**DOI:** 10.2196/65328

**Published:** 2025-08-11

**Authors:** Catherine Knight-Agarwal, Mary Bushell, Mary-Ellen Hooper, Natasha JoJo, Marjorie Atchan, Alison Shield, Angela Douglas, Abu Saleh, Masoud Mohammadian, Irfan Khan, Cheuk Chan, Nico Rovira Iturrieta, Emily Murphy, Tanishta Arza, Deborah Davis

**Affiliations:** 1 Faculty of Health, University of Canberra, 11 Kirinari Street, Bruce, Canberra, 2617, Australia, 61 407806663

**Keywords:** smartphone apps, mobile apps, mHealth, gestational diabetes mellitus, blood glucose level, self-management, smartphone app, mhealth, women's health, diabetes, mellitus, glucose intolerance, hyperglycemia, pregnancy, type 1 diabetes, mobile health application, evidence-based, application, interview, chronic condition, dietary, self-management

## Abstract

**Background:**

Gestational diabetes mellitus (GDM), a type of blood glucose intolerance or hyperglycemia that occurs during pregnancy, is a common condition increasing in prevalence both globally and in Australia. Mobile health apps have been shown to be a useful resource for women with type 1 diabetes and could successfully contribute to GDM management by facilitating healthy behaviors.

**Objective:**

This study aimed to seek the perspectives of health care consumers (HCCs) and health professionals (HPs) regarding the development of a smartphone app for women living with GDM.

**Methods:**

A co-design process with 4 distinct phases underpinned the development of SugarMumma. Phase 1 involved a nonsystematic literature search followed by the creation of an app functions wish list. In phase 2, semistructured interviews with HCCs and HPs were undertaken and then thematically analyzed. In phase 3, a prototype was designed based on social cognitive theory and stakeholder recommendations. Agile project management methodology was used, followed by “user acceptance testing.” During phase 4, a second round of individual interviews was undertaken with HCCs and HPs. The same qualitative methods outlined in phase 2 were used.

**Results:**

In phase 2, individual and didactic interviews were undertaken with HCCs (n=2) and HPs (n=6). Two overarching themes encompassing recommendations for app development emerged: (1) functionality and (2) individualized care. SugarMumma was created in phase 3. Phase 4 involved a second round of individual interviews with HCCs (n=1) and HPs (n=5), resulting in the final theme (3) future directions.

**Conclusions:**

With increasing numbers of people using smartphones, mobile health apps can help manage chronic conditions such as GDM. SugarMumma was designed following extensive stakeholder input. Good functionality, regular notifications, appealing visual aids, positive feedback, relevant dietary advice, and exporting information to HPs are important features to include.

## Introduction

Gestational diabetes mellitus (GDM) is a type of glucose intolerance or hyperglycemia indicated by the onset of elevated blood glucose levels (BGLs) during pregnancy [1]. It is one of the most common pregnancy-related complications, with an increasing prevalence both worldwide and in Australia [2,3]. According to the Australian Institute of Health and Welfare, GDM was diagnosed in 1 out of 6 women who gave birth during 2020‐2021 [4]. Causation is multifactorial, including rising maternal age, high rates of maternal overweight and obesity, and an increasing proportion of women from high-risk ethnic groups giving birth in Australia [1-3,5]. The recent change in diagnostic benchmarks from the previous Australasian Diabetes in Pregnancy Society criteria to those recommended by the International Association of the Diabetes in Pregnancy Study Groups has all contributed to a dramatic increase in the prevalence of GDM in Australia [4,5].

Most Australian public hospitals support a traditional model of care that includes an initial group education class for women diagnosed with GDM facilitated by a credentialed diabetes educator and dietitian, in addition to offering one subsequent individual consultation with either of these health professionals (HPs) and an endocrinologist [4,6,7]. Contemporary best practice management includes (1) lifestyle recommendations such as the consumption of low glycemic index foods, carbohydrate monitoring, appropriate gestational weight gain, BGL, surveillance, and regular physical activity [6,7] and (2) pharmacotherapy encompassing insulin injections or oral hypoglycemic agents such as metformin [7,8]. Through Australia’s National Diabetes Service Scheme, most women diagnosed with GDM have access to a free glucose monitor and subsidized products such as blood glucose strips for the duration of their pregnancy [9].

Nevertheless, due to Australia’s immense land mass, women who reside in remote and rural areas have limited access to health care facilities that offer routine GDM testing in addition to specialist education and support services. The PANDORA (Pregnancy and Neonatal Diabetes Outcomes in Remote Australia) observational study [10] revealed higher rates of maternal diabetes in First Nations women when compared with their non–First Nations counterparts. In this study’s cohort, the GDM and diabetes in pregnancy subgroup experienced poorer birth outcomes with an increased proportion of large for gestational age babies (19% vs 11%) [10]. In 2018, across Australia and New Zealand, Sina et al undertook a thematic analysis of 15 diabetes in pregnancy services. Their findings highlighted not only the difficulty of dealing with increased patient numbers using the traditional model of care but also the complexity of delivering appropriate care for women from culturally and linguistically diverse (CALD) backgrounds [11]. Two recent systematic reviews provide additional support, having revealed that high levels of unmet needs exist in both women from CALD and non-CALD backgrounds [12,13].

Despite BGLs usually returning to normal after parturition, women who experience GDM have a higher risk of developing Type II diabetes, metabolic syndrome, and cardiovascular diseases in the future [14-16]. Furthermore, there is an increased risk of childhood obesity and type II diabetes for their offspring. These outcomes indicate that strategies to optimize GDM management and mitigate potential sequelae are warranted.

There is recognition that mobile health (mHealth) apps could be an important adjunct in GDM management, particularly given the growing evidence for their efficacy in routine type I diabetes care [8]. Several advantages of mHealth apps have been proposed [8]. They enable users to monitor their own health and support them in managing chronic disease independently. In addition, they have been shown to promote healthy behaviors, provide important resources for managing the disease in question (such as type II diabetes), and collect useful health data [17]. Yet, there is a paucity of holistic (encompassing lifestyle recommendations in addition to medication, weight, and BGL monitoring) mHealth apps available for women with GDM. Nevertheless, those that are available mostly include journaling capabilities for glycemic readings and diet [18-21]. Previous research has argued that the most desirable features of mobile apps for GDM self-management involve interactive feedback, credible information, and the capability for integration into existing health care services. Other suggested features include greater personalization or woman-centeredness [22,23].

A recent systematic review looked at the effectiveness of specific mHealth apps for the management of GDM [24]. Very few interventions reported on the clinical effectiveness of GDM-specific mHealth apps [25,26]. The authors reasoned this was predominantly due to both health care consumers (HCCs) and HPs' reluctance in using new technology, difficulty with navigation, and complications with integration into existing health systems [20,21,26-29]. In addition, pharmacotherapy, nutritional, and physical activity outcome data collection and analysis were reported to be lacking [24].

Stakeholder involvement in health research is highly desirable, with national guidelines developed explicitly for this purpose [30]. Stakeholders may include individuals with lived experience of the phenomenon in question, HPs, and academics [31]. This collaborative approach can help identify different perspectives and ideas that would not necessarily be considered by the research team alone. In addition, collaboration between software engineers and the research team is considered vital for ensuring that knowledge from multiple disciplines is used to inform app design and avoid type 1 (where designers do not accommodate user characteristics, preferences, context, or needs) and type 2 (where designers do not accommodate the clinical reality) errors [32].

Therefore, our study aimed to seek the perspectives of HCCs with lived experience of GDM and HPs involved in the care of HCCs with GDM regarding the appropriate information and functions to include in the development of an app for women living with GDM.

## Methods

The SugarMumma app was developed using a co-design process, including 4 distinct phases and supported by an evidence-based approach [33,34].

### Phase 1

The research team included 9 academic clinicians from the University of Canberra’s health disciplines of nutrition and dietetics, midwifery, nursing, pharmacy, and exercise physiology in addition to 3 academics from the faculty of business, government, and law. In late 2022, the research team carried out a nonsystematic literature search of GDM apps available on the market. A paucity of smartphone apps, covering all essential components for the holistic management of GDM, was identified (refer to Multimedia Appendix 1) [29]. Following a round table discussion, the research team put together a “wish list” of functions they would like included in an app of this kind (refer to Multimedia Appendix 2). The team brainstormed potential names for the app and came up with SugarMumma as it conjured happy “sweet” thoughts. In previous years, dietary advice has been heavily focused on eliminating sources of simple carbohydrate, like sucrose, from the diet—an approach that was highly restrictive. Currently, a whole diet approach is recommended where women are encouraged, as part of this approach, to consume carbohydrate foods (including simple sugars) in amounts tailored to the individual [11,12].

### Phase 2

To complement the research team’s “wish list,” a series of HCC and HP consultations were planned. A study information flyer was circulated through the university’s intranet and via the research team’s broader professional networks. Key stakeholders of interest included HCCs with lived experience of GDM and HPs with a current role in GDM management. Following this recruitment drive, didactic interviews (n=3) and individual interviews (n=2) were undertaken online via Teams (Microsoft Corp) in March 2023 at a time that suited participants. CKA, DD, and MEH facilitated these due to their extensive experience as qualitative researchers.

The interview schedule was developed by the research team following a review of the relevant literature [1-4,8,10,12]. Questions revolved around essential features to include in the app and were kept deliberately open, enabling participants to talk with minimum interruption and without judgment. At the end of each interview, participants were given the opportunity to clarify their views and to add any information relating to the topic that may have been missed. All interviews were audio recorded in addition to the lead facilitators (CKA, DD, and MEH) taking handwritten notes. These recordings were then transcribed verbatim by four research assistants (EM, TA, CPJC, and NRI), cross-checked for consistency and entered into a word processing document.

Data were analyzed using a 6-step thematic process as described by Braun and Clarke [35]: (1) familiarization with the data, (2) generating initial codes, (3) interpreting and sorting codes into themes, (4) reviewing themes for coherent patterns, (5) defining and naming the themes, and (6) producing the report. CKA, DD, NRI, EM, CPJC, and TA analyzed all interviews, generating initial codes and discussing the preliminary findings with the broader team. Following this, CKA and NI inserted these codes under relevant themes using an inductive process.

Credibility was addressed by researcher triangulation throughout the analytical process, with CKA, MB, and DD having considerable professional experience working with HCCs with GDM, and MEH having extensive expertise in qualitative research. Feedback from the researchers was discussed at meetings until consensus was reached. The resulting themes, incorporating key recommendations, were then passed on to the app development company.

### Phase 3

The research team engaged an app development company to design the prototype based on information and recommendations from phases 1 and 2. A series of online meetings was undertaken in April and June 2023 between the research team and the app developer to figure out essential and desired features that also heeded budgetary constraints. In addition, it is important to note that social cognitive theory (a behavioral change model) [36] was used as a foundational anchor for the development of SugarMumma. From the end of June 2023 to mid-October 2023, the app developer worked on an initial prototype, suitable for both iOS and Android devices. The React Native framework was determined to be the best “fit” for SugarMumma’s evolution. It is designed to provide a seamless development experience with five key benefits (Table 1).

The Agile project management methodology was then used [37]. App features were developed in short sprints, which enabled individual features to evolve in a modular framework. This process allowed for scalable additions and removals to be completed efficiently [38]. Prior to initiating the second round of key stakeholder feedback, lead research facilitators (DD, MEH, and CKA) undertook their own user acceptance testing (UAT). Testing included checking functionality and ensuring clinical information was accurate. No problems were identified. The final version of SugarMumma and hosting website were ready by early October 2023 for key stakeholder UAT.

**Table 1.** Key benefits of using React Native framework.

Benefits	Description
Cross-platform development	Allows developers to write code once and reuse it across multiple platforms. This means that developers can use the same codebase to create apps for both iOS and Android, saving significant development time and effort.
Faster development	Uses a “hot reloading” feature that enables developers to see the changes they make to the code in real time. This feature speeds up the development process, as developers can quickly identify and fix issues as they arise.
Cost-effective	Allows developers to write code once and use it across multiple platforms, it significantly reduces the cost of development. This is because it eliminates the need for developers to write separate codebases for each platform.
Large developer community	It has a large developer community, which means there are plenty of resources, tools, and libraries available to help developers build mobile apps. This community also provides ongoing support and updates, making it easier for developers to stay up to date with the latest technologies and best practices.
Native performance	Uses native components, which means that the resulting mobile app has the same performance and user experience as a natively developed app.

### Phase 4

HCCs and HPs from phase 2 were notified by email in mid-October 2023 about the completion of SugarMumma’s initial prototype and, if they agreed to continue with their involvement, were given 7 days to access the app. They were encouraged to operate the app as if it were for their own use. Following the UAT, individual interviews with an HCC (n=1) and HPs (n=5) were undertaken with the same methods as described in phase 2. The only difference was during data analysis where a more deductive approach was taken compared to phase 3, as the aim was to review the actual app (not simply a wish list) and to ascertain if it met HCCs' needs. Finally, recommendations were passed on to the app company for consideration in future development of SugarMumma.

### Ethical Considerations

This study was supported by funding from the Australian Digital Translation Fund. Before each phase of this study, participants (both HCCs and HPs) gave informed written consent. Ethical approval was granted by the University of Canberra’s Human Research Ethics Committee (reference 202311994). All participant details were kept confidential with pseudonyms used to ensure anonymity. HCCs each received a compensation of Aus $100 (US $64.87). HPs provided their time free of charge.

## Results

### Overview

See Table 2 for participant characteristics.

Data analysis revealed two themes resulting from phase 2: functionality and individualized care. Interviews had an average duration of 45 minutes each (both didactic and individual).

**Table 2.** Demographics of HCCs and HPs.

Characteristics	Participants, n
**Health professionals, sex**	
**Sex**	
Male	1
Female	5
**Age (years)**	
25‐44	1
45‐65	5
**Experience of working in GDM^a^ (years**)	
0‐5	2
5‐10	2
>10	2
**Occupation**	
Endocrinologist	1
Diabetes nurse educator	2
Midwife	1
Obstetrician	1
Dietitian	1
**Healthcare consumers, previous experience**	
Recent experience living with GDM	2

a GDM: gestational diabetes mellitus.

### Phase 2: Qualitative Interviews

#### Functionality

Participants emphasized the need for SugarMumma to be a patient-centered, informative app that could:

*… remind women about appointments* [HP1] in addition to including *information resources that are reliable and educational… (these) would be very useful* [HP1]. The app also needed to be GDM-specific: *I never even heard of any GDM specific apps (when I was pregnant) … I didn’t use one* [HCC2] and *No HP suggested any to me either.*

[HCC1]

Similarly, other HCCs claimed that SugarMumma should aim to make life less challenging:

*I was told to record all my diet and BGLs on an A4 piece of paper…. I was terrible at that. I would have loved a smartphone app* [HCC2]. The desired capacity for an app to easily *record ones BGLs* [W1] led another HCC to add *that graphing BGLs …… would be interesting and useful.*

[HCC2]

The value of providing HCCs with evidence-based guidance was acknowledged by HPs:

*… most of the ones I use for my people are really around carb counting and insulin dosing… so they (the women) put in what they are eating, and it tracks everything…* [HP2] in addition to *getting them to do a (food and BGL) diary, we try to do whatever we can to get some information on them…*

[HP2]

It was emphasized that the app should ideally be compatible with various devices so that all potential users can download it:

*…a lot of them (patients) have OPPO phones, you know most of the apps are only compatible with Samsung or iPhone…*

[HP2]

Ultimately, it was felt that SugarMumma should be straightforward to use to cater for the diverse needs of Australia’s multicultural society as one HP pointed out:

... we have a large proportion (of women) with very low health literacy. So, it (needs to) be simple for them.

[HP2]

Both the HCCs and HPs felt SugarMumma would make coordination of maternity care easier:

*I think it would be very useful to pass information on to my doctor and being added to the record…. I remember the nurse coming in and looking at the book (for BGL’s) and then typing (the levels) into the computer and saying do you remember exactly what number that was when you scribbled it in…*.

[HCC1]

Communicating GDM-related health data between an HCC and her HP team via SugarMumma was seen as an important feature:

*Maybe none of your figures (BGL’s) may be concerning, like a single one, however there could be a pattern, and so you don’t know that and you may not have an appointment but something that triggers you to contact the doctor or them to get in touch…… especially if you are not close (in proximity) to doctors.*

[HCC1]

In addition, it was suggested that SugarMumma should enable the consensual sharing of data:

*for research or whatever but also knowing it can be anonymised…… because if women are collecting all this data, it is useful to have in terms of what else can be done.*

[HCC1]

In some situations, it was recognized that communication between a HCC and their HP via SugarMumma may be less stressful than a face-to-face meeting:

*A lot of them (women) struggle with what their glucose levels are doing... they don’t want to show us what is really happening despite usually being motivated (to follow recommendations).*

[HP2]

#### Individualized Care

It was recognized that a user-friendly app, tailored to the individual, could empower self-management especially when there was no immediate need for HP intervention:

*...(for example, to) take the load off….. knowing when you might be high in insulin (and then should eat something)…. or when there is the need for a bit of prepreparation if you are going out for dinner and require more insulin.*

[HCC1]

Up-to-date information that is constantly available and easily accessible was very appealing, with one of the HCCs claiming: “Meal plans would be very helpful to include” (in SugarMumma; HCC2). In addition, a range of foods and recipes that were not just*: “*Anglo focused” (HCC1). The need for more culturally appropriate inclusions, particularly for populations with challenging engagement patterns, was highlighted:

*We have the large islander population, we have (an) aboriginal population, and not to generalize too much but our islander population is probably the most challenging because they really don’t engage with healthcare and don’t see the value in it*.

[HP2]

HCCs acknowledged that SugarMumma could motivate individual users to monitor other important variables relevant to pregnancy in addition to BGLs, for example:

*It is really good to help track weight…. I found the information (available from HPs) quite confusing…. I was in that overweight category when I got pregnant…… I didn’t put on much during my pregnancy and there wasn’t much information about if you are in that middle bit……and how normal is that as I knew the baby was growing but particularly when you are having your first pregnancy you’re worried about everything….. like should I be putting on weight…. Knowing what is normal…. And knowing what information is relevant is very important to include.*

[HCC1]

The importance of physical activity for the management of BGLs was emphasized by HPs who proposed integrating features that: “Encourage all our women to go for a 10 min walk after they eat” (HP2). A University branded app was believed to be credible, thus a motivator for its use: “Yeah, I think it’s really good that it’s coming from like a tertiary institution” (HCC2).

#### Phase 3: App Development

SugarMumma was developed with BGL, insulin, diet, physical activity, and weight tracking functions in addition to providing glycemic and weight gain targets. A facility to record food intake concentrating on carbohydrates is a key feature. Links to government-approved websites on topics such as pregnancy-related nutrition and exercise were also included. SugarMumma provides medication reminders and the ability to generate reports if desired by HCCs (refer to MultimediaAppendices 3  4).

#### Phase 4: Qualitative Interviews

Greater use of photos and icons instead of extensive text was suggested as one way to enhance user friendliness of the initial prototype: “…maybe something like pictures…… to click on for what they (HCCs) want to use rather than lot of words” (HP2) as well as “a lot of dropdowns rather than lots of free text… like just the ease of use I guess” (HP4). Whereas other HPs expressed a preference for manual input of numerical data: “I didn’t particularly like scrolling through the numbers... I’d rather type in what my number is” (HP3).

Likewise, HPs highlighted the need for SugarMumma to send more supportive, individualized messages especially when there are extended periods of time between antenatal appointments:

*I just sort of wish there was something* (in the app)….. *just a little prompt to say it has been noticed you’ve got this many highs and this range, therefore maybe consider contacting your diabetes healthcare provider or something like that.*

[HP2]

HCCs generally found SugarMumma to be useful compared to a paper book for communicating lifestyle and BGL data. However, frustration was voiced around how food was encouraged to be recorded: “I wouldn’t necessarily know the grams of something I’ve eaten” (HCC2).

HPs discussed the challenge of setting specific fasting targets which may differ from state to state and from HCC to HCC and not simply include generic glycemic recommendations: “I worry sometimes when you tell some of the women a specific number, they get quite hung up on that number” (HP3). It was also suggested that not using continuous BGL measurements but the provision of guidance on “what to do if it is too high (BGL)…., and where to go for help (if this happens)” (HP5). A further suggestion was to make SugarMumma multilingual to accommodate the diverse range of ethnicities as: *“*not everybody has English as the first language either…” (HP3).

It recognized that an app for GDM management could include information relevant to the postpartum period and beyond:

*They also talk about your kids getting it (diabetes) and what to look out for later and even in terms of the data if you can download it and then email it to your doctors……*

[HCC2]

Ultimately, it was recognized that for SugarMumma to be effective, it “must be allowed to integrate into the existing health systems” (HP6).

## Discussion

### Principal Findings

An extensive search of the peer-review literature in addition to qualitative interviews with HCCs and HPs informed development of SugarMumma, a smartphone app for the management of GDM. Key considerations were the importance of functionality and individualized care. Our findings are consistent with similar work of this kind [29].

### Comparison With Previous Work

Good functionality, reliable information in addition to key utilities such as BGL monitoring, appointment reminders, and ability to generate health reports if desired are key features of SugarMumma. Similarly, Garnweidner-Holme et al [25] stressed the importance of a simple and easy-to-navigate menu design for pregnancy-related apps. Nevertheless, in phase 4, HPs suggested the inclusion of more dropdown menus and less text, believing this would enhance user-friendliness.

Safiee et al [29] previously identified that app first impressions are provoked by the simplicity of language and attractive visuals. Likewise, participants in our study highlighted a preference for images and icons over words to facilitate easy interpretation of BGLs, medications, exercise, and weight data (with relevant feedback) [29,39-41].

Previous studies have reported some women’s difficulty in remembering the copious amounts of information provided during antenatal care appointments [19,29,42]. A “one-stop shop” of credible resources to assist women in managing GDM is one of the proposed benefits of SugarMumma [41,42]. A qualitative study of mHealth apps identified that users rely on recommendations from a wide range of sources including strangers, friends, authoritative figures including HPs, and recognizable branding to inform app selection [42-44]

Frontline intervention for GDM management requires dietary monitoring [45,46]. Electronic food diary systems provide an opportunity for improved self-management, beneficial to both HCCs with GDM and HPs [46]. Our study indicated some participants considered carbohydrate monitoring to be a more important feature than BGL surveillance. Nevertheless, the measurement of food in grams was reported to be confusing. This correlates with similar work by Safiee et al [29], where they found that participants preferred measurements in bowls or teaspoons over dimensions in grams [29].

Participants in our study believed the generation of multifactorial reports (eg BGL or Food or Activity) to be an important function along with notetaking with the ability to contextualize data [44,46]. Similar literature has highlighted a desire for direct in-app communications with HPs, which is currently lacking in many diabetes apps [44,47]. Falsification in reporting BGLs due to HCCs embarrassment or for the purpose of receiving positive feedback has been acknowledged previously [19,29]. Automatic but consensual transfer of data from HCCs' mobile phones to their HP may negate this risk.

Visual cues are a powerful tool in engaging user experience, with consideration to culture and varying degrees of health literacy. Images may assist culturally and linguistically diverse users to navigate the app where their preferred language is not available and has the potential to diminish feelings of social exclusion. Inclusivity may also be fostered by adjusting illustrations to be appropriate for different ethnic groups [42]. Feedback from the initial prototype of SugarMumma described the overall design and language largely appropriate for the target audience. Nevertheless, it is currently only available in English.

Access to service provision for GDM management can be challenging based on factors including rurality or competing demands. These challenges are compounded by the overwhelming and drastic increase in the frequency of antenatal consultations associated with a GDM diagnosis [45,48]. HCCs and HPs alike identify the challenges in accessing health services for GDM management, including absence from work, reduced income, travel, and inconvenience (particularly if parenting other children) [29,48]. The use of smartphone apps such as SugarMumma as an adjunct for GDM management may help decrease travel time, minimize unnecessary appointments, reduce absence from work, and potentially lower stress [49,50].

### Future Directions

Our research has not only informed the initial stages of app development but also provided a pathway for further refinements. mHealth has an increasingly complementary role to current GDM management interventions, in the context of a limited number of HPs and an increasing number of GDM diagnoses [29]. The SugarMumma app has been designed with the long-term goal of seamless integration with existing documentation systems, to be used as an adjunctive tool to standard GDM interventions. When introduced immediately following diagnosis, HPs perceive similar GDM management apps as appropriate tools for management [40,41].

Women receive a significant amount of support and monitoring during their pregnancy; however, both HCCs and HPs suggest that this level of support and follow-up is often abandoned postbirth [45]. Considering the impact of the baby’s health on motivation for GDM management, loss of motivation is unsurprising after birth [19]. We hope to expand the functionality of the SugarMumma app to include provision for women during the postnatal period by highlighting the risk of type 2 diabetes mellitus and importance of ongoing screening.

There are some limitations that must be acknowledged. This study contained only a small sample size of HCCs and HPs, thus limiting the rigor of our results. We acknowledge that our participants' views may not reflect those living with GDM or caring for HCCs with GDM elsewhere. Most of our participants were female HPs without lived experience of GDM. This may have influenced the support needs and app functions emphasized in our findings. The small number of interviews was due to participant scheduling constraints. It is also important to point out that the SugarMumma app has been designed as an adjunct to usual maternity care and in no way should replace the individual advice provided by a HP.

### Conclusion

With the increased number of people using smartphones in their daily lives, mHealth apps are being considered as a novel way to manage chronic conditions such as GDM. This study has revealed that key stakeholders appreciate features including simplicity, regular notifications, appealing visual aids, and positive feedback. Future work is planned to enhance the current prototype.

## Supplementary material

10.2196/65328 Multimedia Appendix 1 Summary of the currently available gestational diabetes mellitus apps in the market.

10.2196/65328 Multimedia Appendix 2 The “wish list”—what academic health clinicians would you like to see in a holistic app for women living with gestational diabetes mellitus?

10.2196/65328 Multimedia Appendix 3 SugarMumma screenshot (I).

10.2196/65328 Multimedia Appendix 4 SugarMumma screenshot (II).

## Abbreviations

BGL: blood glucose level
CALD: culturally and linguistically diverse
GDM: gestational diabetes mellitus
HCC: health care consumer
HP: health professional
mHealth: mobile health
PANDORA: Pregnancy and Neonatal Diabetes Outcomes in Remote Australia
UAT: user acceptance testing

## References

[1] Mirghani Dirar A, Doupis J (2017). Gestational diabetes from A to Z. World J Diabetes.

[2] McIntyre HD, Catalano P, Zhang C, Desoye G, Mathiesen ER, Damm P (2019). Gestational diabetes mellitus. Nat Rev Dis Primers.

[3] Laurie JG, McIntyre HD (2020). A review of the current status of gestational diabetes mellitus in Australia-the clinical impact of changing population demographics and diagnostic criteria on prevalence. Int J Environ Res Public Health.

[4] Behboudi-Gandevani S, Amiri M, Bidhendi Yarandi R, Ramezani Tehrani F (2019). The impact of diagnostic criteria for gestational diabetes on its prevalence: a systematic review and meta-analysis. Diabetol Metab Syndr.

[5] Chen P, Wang S, Ji J (2015). Risk factors and management of gestational diabetes. Cell Biochem Biophys.

[6] Rasmussen L, Poulsen CW, Kampmann U, Smedegaard SB, Ovesen PG, Fuglsang J (2020). Diet and healthy lifestyle in the management of gestational diabetes mellitus. Nutrients.

[7] Nankervis A, Price S, Conn J (2018). Gestational diabetes mellitus: A pragmatic approach to diagnosis and management. Aust J Gen Pract.

[8] Chomutare T, Fernandez-Luque L, Arsand E, Hartvigsen G (2011). Features of mobile diabetes applications: review of the literature and analysis of current applications compared against evidence-based guidelines. J Med Internet Res.

[9] (2024). Blood glucose monitoring National Diabetes Services Scheme. National Diabetes Services Scheme.

[10] Maple-Brown L, Lee IL, Longmore D (2019). Pregnancy and neonatal diabetes outcomes in remote Australia: The PANDORA study-an observational birth cohort. Int J Epidemiol.

[11] Sina M, Cade TJ, Flack J (2020). Antenatal models of care for women with gestational diabetes mellitus: Vignettes from an international meeting. Aust NZ J Obst Gynaeco.

[12] Davis D, Kurz E, Hooper ME (2024). The holistic maternity care needs of women with gestational diabetes mellitus: A systematic review with thematic synthesis. Women Birth.

[13] Tzotzis L, Hooper ME, Douglas A (2023). The needs and experiences of women with gestational diabetes mellitus from minority ethnic backgrounds in high-income nations: A systematic integrative review. Women Birth.

[14] Damm P (2009). Future risk of diabetes in mother and child after gestational diabetes mellitus. Intl J Gynecology Obste.

[15] Reece EA, Leguizamón G, Wiznitzer A (2009). Gestational diabetes: the need for a common ground. Lancet.

[16] Sullivan SD, Umans JG, Ratner R (2012). Gestational diabetes: implications for cardiovascular health. Curr Diab Rep.

[17] Kumar S, Nilsen WJ, Abernethy A (2013). Mobile health technology evaluation: the mHealth evidence workshop. Am J Prev Med.

[18] Daley BJ, Ni’Man M, Neves MR (2022). mHealth apps for gestational diabetes mellitus that provide clinical decision support or artificial intelligence: A scoping review. Diabet Med.

[19] Skar JB, Garnweidner-Holme LM, Lukasse M, Terragni L (2018). Women’s experiences with using a smartphone app (the Pregnant+ app) to manage gestational diabetes mellitus in a randomised controlled trial. Midwifery.

[20] Seely EW, Weitzman PF, Cortes D, Romero Vicente S, Levkoff SE (2020). Development and feasibility of an app to decrease risk factors for type 2 diabetes in Hispanic women with recent gestational diabetes (Hola Bebé, Adiós Diabetes): Pilot pre-post study. JMIR Form Res.

[21] Varnfield M, Redd C, Stoney RM (2021). M♡THer, an mHealth system to support women with gestational diabetes mellitus: Feasibility and acceptability study. Diabetes Technol Ther.

[22] Hewage S, Audimulam J, Sullivan E, Chi C, Yew TW, Yoong J (2020). Barriers to gestational diabetes management and preferred interventions for women with gestational diabetes in singapore: Mixed methods study. JMIR Form Res.

[23] Kytö M, Koivusalo S, Ruonala A (2022). Behavior change app for self-management of gestational diabetes: Design and evaluation of desirable features. JMIR Hum Factors.

[24] Eberle C, Loehnert M, Stichling S (2021). Effectivness of specific mobile health applications (mHealth-apps) in gestational diabtetes mellitus: a systematic review. BMC Pregnancy Childbirth.

[25] Garnweidner-Holme L, Henriksen L, Torheim LE, Lukasse M (2020). Effect of the Pregnant+ Smartphone app on the dietary behavior of women with gestational diabetes mellitus: Secondary analysis of a randomized controlled trial. JMIR Mhealth Uhealth.

[26] Borgen I, Småstuen MC, Jacobsen AF (2019). Effect of the Pregnant+ Smartphone application in women with gestational diabetes mellitus: a randomised controlled trial in Norway. BMJ Open.

[27] Surendran S, Lim CS, Koh GCH, Yew TW, Tai ES, Foong PS (2021). Women’s usage behavior and perceived usefulness with using a mobile health application for gestational diabetes mellitus: Mixed-methods study. Int J Environ Res Public Health.

[28] Wake DJ, He J, Czesak AM, Mughal F, Cunningham SG (2016). MyDiabetesMyWay: An evolving national data driven diabetes self-management platform. J Diabetes Sci Technol.

[29] Safiee L, Rough DJ, Whitford H (2022). Barriers to and facilitators of using eHealth to support gestational diabetes mellitus self-management: Systematic literature review of perceptions of health care professionals and women with gestational diabetes mellitus. J Med Internet Res.

[30] (2024). Consumer and community engagement. The National Health and Medical Research Council.

[31] Berry N, Lobban F, Bucci S (2019). A qualitative exploration of service user views about using digital health interventions for self-management in severe mental health problems. BMC Psychiatry.

[32] Blandford A, Gibbs J, Newhouse N, Perski O, Singh A, Murray E (2018). Seven lessons for interdisciplinary research on interactive digital health interventions. Digit Health.

[33] Knight-Agarwal C, Davis DL, Williams L, Davey R, Cox R, Clarke A (2015). Development and Pilot Testing of the Eating4two Mobile Phone App to Monitor Gestational Weight Gain. JMIR Mhealth Uhealth.

[34] Bechard LE, Wei C, Tupling O, Keller H, McAiney CA, Middleton LE (2021). Evaluating the co‐design of a program to supporting living well with dementia. Alzheimers Dement.

[35] Braun V, Clarke V (2006). Using thematic analysis in psychology. Qual Res Psychol.

[36] Schunk DH (2012). APA Educational Psychology Handbook, Vol 1: Theories, Constructs, and Critical Issues.

[37] Winter RJ (2014). Agile software development: Principles, patterns, and practices. Perf Improv.

[38] Mohd C, Shahbodin F (2015). Personalized learning environment: Alpha testing, beta testing & user acceptance test. Procedia Soc Behav Sci.

[39] Garnweidner-Holme LM, Borgen I, Garitano I, Noll J, Lukasse M (2015). Designing and developing a mobile smartphone application for women with gestational diabetes mellitus followed-up at diabetes outpatient clinics in Norway. Healthcare (Basel).

[40] Al Hashmi I, Alsabti H, Al Omari O, Al Nasseri Y, Khalaf A (2022). Development, feasibility and acceptability of a self-efficacy-enhancing smartphone application among pregnant women with gestational diabetes mellitus: single- arm pilot clinical trial. BMC Pregnancy Childbirth.

[41] Duan B, Liu Z, Liu W, Gou B (2022). Assessing the views and needs of people at high risk of gestational diabetes mellitus for the development of mobile health apps: Descriptive qualitative study. JMIR Form Res.

[42] Garnweidner-Holme L, Hoel Andersen T, Sando MW, Noll J, Lukasse M (2018). Health care professionals’ attitudes toward, and experiences of using, a culture-sensitive smartphone app for women with gestational diabetes mellitus: Qualitative study. JMIR Mhealth Uhealth.

[43] Peng W, Kanthawala S, Yuan S, Hussain SA (2016). A qualitative study of user perceptions of mobile health apps. BMC Public Health.

[44] Sushko K, Menezes HT, Wang QR, Nerenberg K, Fitzpatrick-Lewis D, Sherifali D (2023). Patient-reported benefits and limitations of mobile health technologies for diabetes in pregnancy: A scoping review. Can J Diabetes.

[45] Parsons J, Sparrow K, Ismail K, Hunt K, Rogers H, Forbes A (2018). Experiences of gestational diabetes and gestational diabetes care: a focus group and interview study. BMC Pregnancy Childbirth.

[46] Pais S, Petrova K, Parry D (2022). Enhancing system acceptance through user-centred design: Integrating patient generated wellness data. Sensors (Basel).

[47] Ali Sherazi B, Laeer S, Krutisch S, Dabidian A, Schlottau S, Obarcanin E (2023). Functions of mHealth diabetes apps that enable the provision of pharmaceutical care: Criteria development and evaluation of popular apps. IJERPH.

[48] Rasekaba T, Nightingale H, Furler J, Lim WK, Triay J, Blackberry I (2021). Women, clinician and IT staff perspectives on telehealth for enhanced gestational diabetes mellitus management in an Australian rural/regional setting. Rural Remote Health.

[49] Campbell S, Roux N, Preece C (2017). Paths to improving care of Australian Aboriginal and Torres Strait Islander women following gestational diabetes. Prim Health Care Res Dev.

[50] Sun S, Pellowski J, Pisani C (2023). Experiences of stigma, psychological distress, and facilitative coping among pregnant people with gestational diabetes mellitus. BMC Pregnancy Childbirth.

