# Supplementary material for: Development of a Smartphone App for Women Living With Gestational Diabetes Mellitus: Qualitative Study
Source: JMIR Diabetes. 2025 Aug 11;10:e65328. doi: 10.2196/65328 (PMC12338752; doi:10.2196/65328)
Supplement: Multimedia Appendix 1 [file diabetes-v10-e65328-s001.docx]

**Appendix 1**:Summary of the currently available GDM apps in the market

|  | **Name (Company)** | **Features** | **Weaknesses** |
| --- | --- | --- | --- |
| 1 | Diabetes Forum  (DDM Health) | Discussion forum | No other functionality |
| 2 | Gestational Diabetes  (Rotem Maoz) | Nutrition log  Blood glucose tracking | Limited functionality |
| 3 | Best Gestational Diabetes Info  (tomtomdev) | Education/Information | Limited functionality  No interaction |
| 4 | Pregnant with Diabetes  (heyworld.dk) | Education/Information | Limited functionality  No interaction |
| 5 | Gestational Diabetes Health Tips and Care  (Gippy) | Education/Information | Limited functionality  No interaction |
| 6 | Gestational Diabetes Meal Plan  (DigitalifyApps) | Education/Information | Limited functionality  No interaction |
| 7 | Gestational Diabetes Stress Scale  (Anandhasayanam Aravamuthan) | Scale | Limited functionality |
| 8 | GDm-Helath  (Sensyne Health) | Blood glucose tracking  Education/Information | Limited functionality |
| 9 | M♡THer  (CSIRO) | Education/Information  Blood glucose tracking  Diet and Exercise log  Other metrics (BP, Weight)  Reports/data sharing | Limited functionality  No integration |
| 10 | MyFetalLife  (Fetal LifeApp) | Pregnancy calculator  Kick, contraction counter  Blood glucose tracking  Other metrics (BP, Weight)  Reports/Data Sharing | Broad focus, not GDM specific  Limited functionality |
| 11 | GlucoKeeper  (iHealth Labs Inc.) | Blood glucose tracking  Reports/Data sharing | All types of diabetes, not GDM specific  Limited functionality |
| 12 | Glucolyf  (Ingenuity Insights) | Blood glucose tracking  Diet and exercise log  Bolus Insulin calculator  Reports/Data Sharing Integration: Dexcom CGM, Fitbit | All types of diabetes, not GDM specific |
| 13 | gluQUO  (QUO Health SL) | Blood glucose tracking  Diet and exercise log  Bolus insulin calculator  Reports/Data sharing  Integration: Apple Health | All types of diabetes, not GDM specific  Subscription |
| 14 | MySugr  (mySugr GmbH) | Blood glucose tracking  Diet and exercise log  Bolus insulin calculator  Reports/Data sharing  Integration: CGM, Accu chek (Blood glucose check), Google Fit | All types of diabetes, not GDM specific  Many functions only available on premium version by subscription |
| 15 | Glucose Buddy Diabetes Tracker  (Azumio, Inc.) | Blood Glucose tracking  Diet and exercise log  Other metrics (BP, weight)  Bolus Insulin Calculation  Reports/Data sharing  Education/Information  Integration: Dexcon CGM, Apple Health | All types of diabetes, not GDM specific  Many functions only available on premium version by subscription |
