# Supplementary material for: Development of a Smartphone App for Women Living With Gestational Diabetes Mellitus: Qualitative Study
Source: JMIR Diabetes. 2025 Aug 11;10:e65328. doi: 10.2196/65328 (PMC12338752; doi:10.2196/65328)
Supplement: Multimedia Appendix 2 [file diabetes-v10-e65328-s002.docx]

**Appendix 2:** The ‘wish list’ – What academic health clinicians would like to see in a holistic app for women living with GDM

- A gestational weight gain tracker with individual guidance on optimal weight gain.
- Facility to log intermittent blood glucose readings and the option of integration with continuous glucose monitoring devices for real time readings of blood glucose levels.
- Facility to log physical activity and the option of integration with activity tracking devices.
- Facility to log carbohydrate exchanges/portions and the option of integration with other registered apps MyFitnessPal™ to record nutritional intake.
- Recommendations for nutrition in pregnancy based on the Nutrient Reference Values of Australia. Link to: [Nutrient Reference Values | Eat For Health](https://www.eatforhealth.gov.au/nutrient-reference-values)
- Recommendations for physical activity in pregnancy. Link to: [Guidelines for physical activity during pregnancy](https://www.health.gov.au/sites/default/files/2023-11/physical-activity-and-exercise-during-pregnancy-guidelines-brochure.pdf)
- Graphs which provide trend data in blood glucose levels, mapped against diabetes medications, carbohydrate intake and physical activity.
- Facility to generate reports for health professionals if approved/desired by HCCs e.g. weekly insulin mapping against carbohydrate intake and physical activity.
