# Supplementary figures and images for: Development of a Smartphone App for Women Living With Gestational Diabetes Mellitus: Qualitative Study
Source: JMIR Diabetes. 2025 Aug 11;10:e65328. doi: 10.2196/65328 (PMC12338752; doi:10.2196/65328)

**Appendix 3:** SugarMumma screenshot


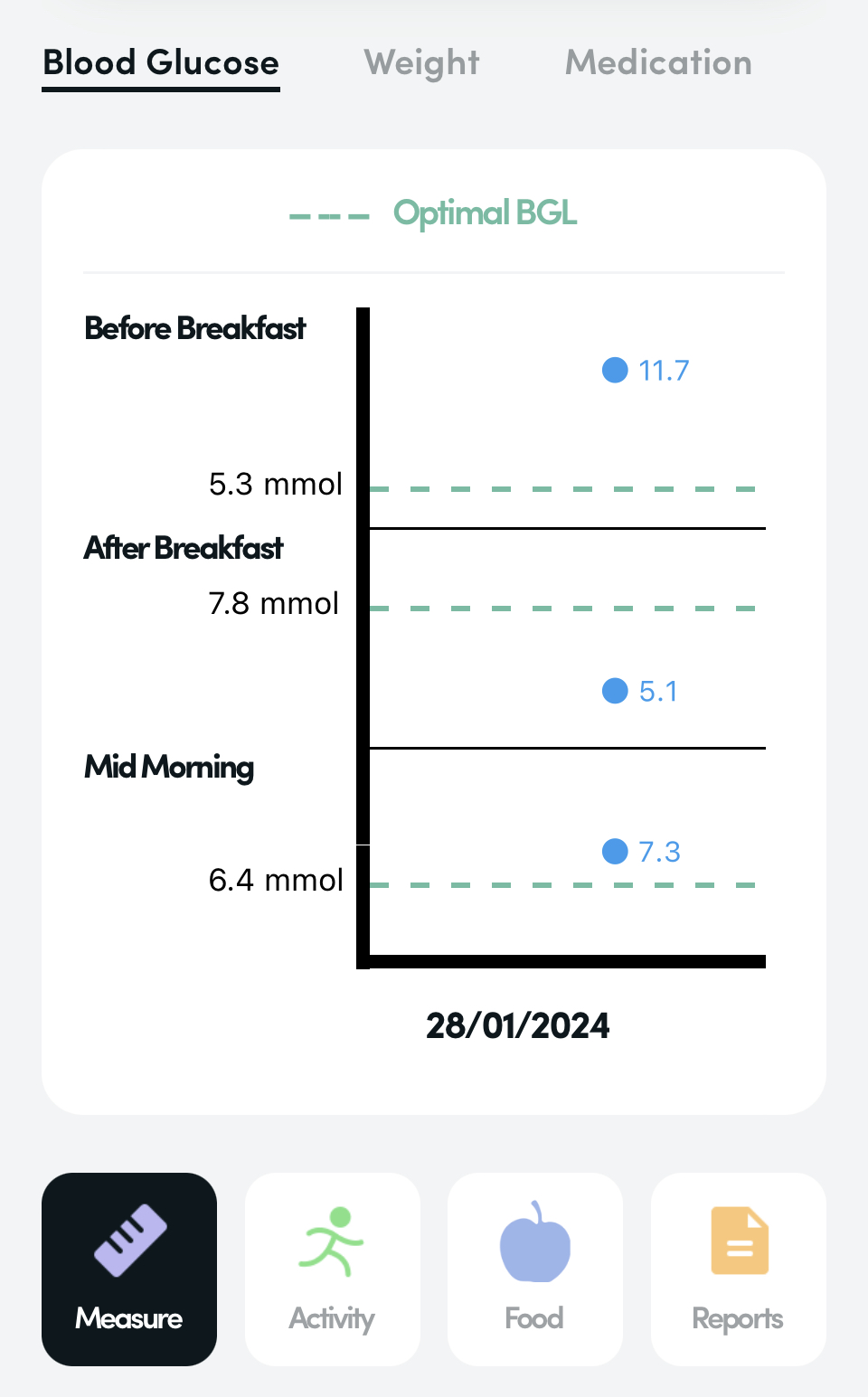

Supplement: Multimedia Appendix 3 [file diabetes-v10-e65328-s003.docx]

**Appendix 4:** SugarMumma screenshot


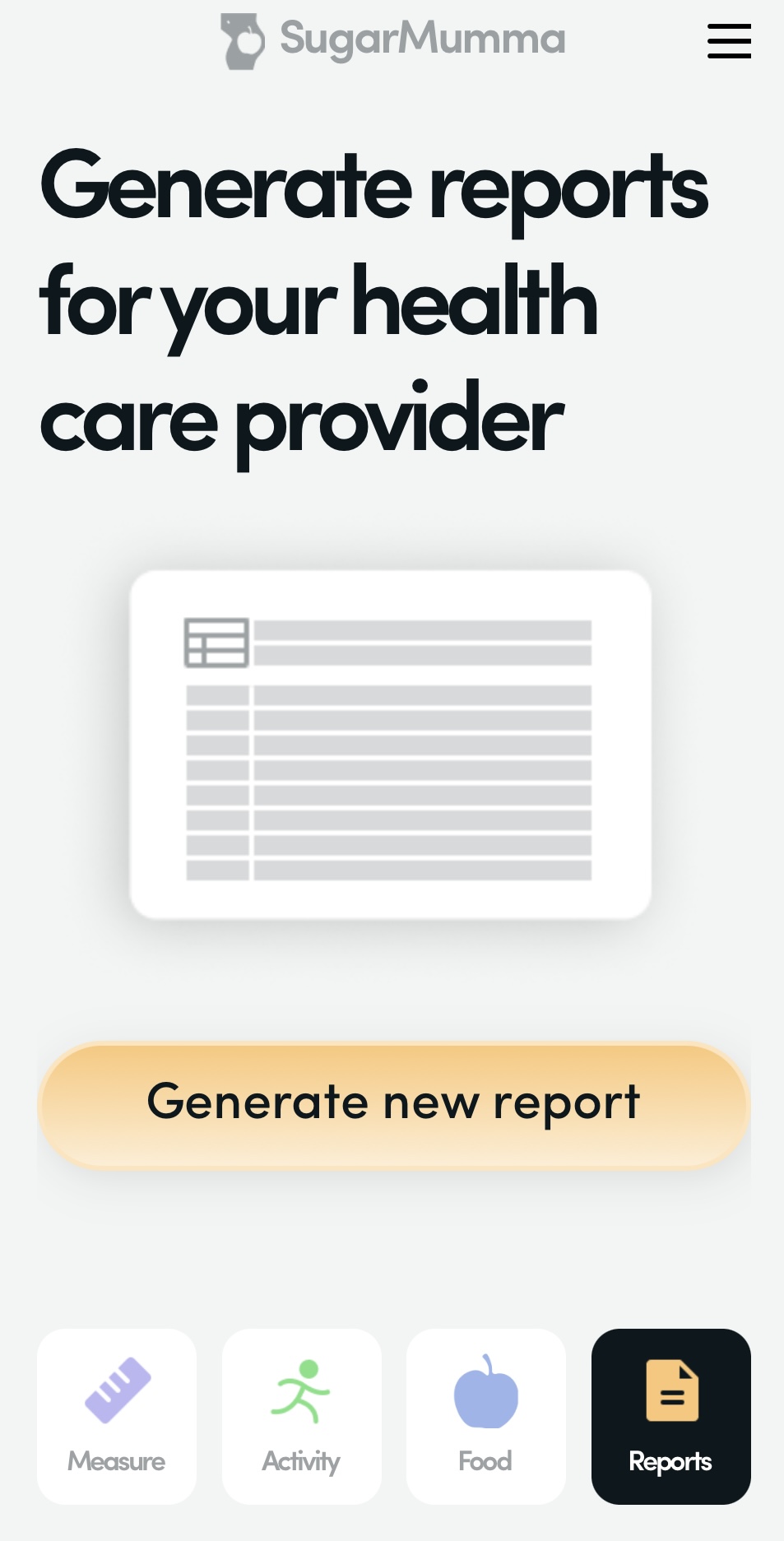

Supplement: Multimedia Appendix 4 [file diabetes-v10-e65328-s004.docx]
